# Reports of Societies

**Published:** 1874-10

**Authors:** 


### Reports of Societies.

***Chicago Society of Physicians and Surgeons.*** *Transactions at Regular Meeting, August* 24, 1874. Reported by RALPH E. STARKWEATHER, M.D.

Dr. John Bartlett, President, in the chair. The Secretary being absent, Dr. F. H. Davis was chosen Secretary *pro tempore.* Dr. F. L. Wadsworth received an unanimous election to the membership of the Society.

Having called Dr. Hamill to the chair, Dr. Bartlett read a report of a case of eclampsia in a pregnant woman, due to uræmia, which was of rare interest, and elicited a very able and animated discussion, sustained by numerous members of the Society. The following is an abstract of the case:

Mrs. M., now twenty-five years of age, began to menstruate at the age of twelve. Her first menstruation alarmed her, and was looked upon as being something dreadful. Rising at night, she went to a well and washed her clothing, bedding and person. Since that event, every period has been one of so much pain and nervous disturbance, that she has regarded herself as having a serious uterine disease. She was liable to hysterical paroxysms upon the approach of her periods, before marriage. At the age of fifteen, while traveling, she went from morning till night without micturating; and upon then attempting so to do, she was unable to accomplish the act. The retention continued for two days longer, until relieved by catheterism.

This was followed by a sickness of several weeks, the nature of which is unknown; but ever afterwards, at irregular intervals, she was liable to fainting turns. These attacks began with palpitation of the heart, indistinctness of vision, a state of semi-consciousness, and of sinking away, as if dying.

In other respects her general condition was excellent. Marrying at the age of eighteen, a year later she gave birth, at the eighth month, to a fœtus that had been dead some weeks. The delivery seems to have been followed by a condition of septicæmia.

The vomiting of the two pregnancies which followed, was unusually severe and prolonged. In June, Dr. Bartlett was called to attend the patient in her fourth parturition. She had uterine pains, intense headache, amblyopia, occasional delirium, great irritability of the bladder, and œdema of the face and hands. The uterine pain was regarded as false. Uræmia was predicted, and the friends notified of possible grave accidents. Wine of colchicum seed and morphia were ordered. The colchicum was given for more than a week, with the effect of removing the cerebral symptoms above named.

July 25, the following was the condition of the patient in the first stage of labor:

Considerable œdema of the face and throat, conjunctivæ conjested; throbbing headache, especially on the right side; intense epigastric pain, described as if the stomach and womb were being torn apart. Morphine diminished somewhat the pain in the head and epigastrium.

The second stage of labor, lasting three hours, terminated at 2 A. M., July 26, by the delivery of a male child, weighing less than four pounds. The third stage terminated in an hour; one hour thereafter the patient complained of noises in the room when there were none, and could not see the cup, containing chloral, offered her. A convulsion immediately followed, succeeded by stupor, during which the patient was bled to ten ounces, and one drachm of chloral was injected into the bowels. In one hour a *second* spasm occurred. A purgative enema was given, and a scruple of chloral administered. Upon consultation with Dr. Byford, it was determined to deplete still farther, and some twenty-five ounces of blood were withdrawn. Croton oil was administered; and eighteen grains of chloral ordered to be given every half hour, till several doses had been taken. A *third* convulsion followed, three hours after the second attack. It was found that neither by the stomach nor rectum would chloral be retained. During the day, convulsions occurred about every three hours, the patient lying stupid during the intervals, but able to be aroused. Toward night the tendency to spasm was increased, so that an attempt to give medicines by the mouth or rectum, or by the hypodermic syringe, or the exhibition of chloroform, was liable to bring on spasms. There was now occasionally an active, wild delirium. By taking advantage of the stupor following a convulsion, a quantity of morphine was passed under the skin. To increase or renew the effects of morphia, it became necessary to give chloroform, in order successfully to make use of the hypodermic injection. The favorable and satisfactory effect of this medicine was maintained for fifty-five hours; small quantities of water and milk being the only nourishment.

Iced cloths were kept constantly to the head, and during the paroxysms masses of ice were applied to the scalp; serious congestion of the face never occurred.

The urine drawn by catheter on the first day, was found to be one-half albuminous. On the third day it was clear, and not albuminous.

The convulsions returned at the time the milk-fever was to be expected, but were, for several days, of moderate severity. The first series of convulsions were severe, accompanied by insensibility, and followed by stupor. The second were, for a time, milder; the patient retained consciousness, breathed freely, and conversed while convulsed. This second series was thought to have a hysterical element in them. The first series was regarded as those of true eclampsia depending upon *the so-called uræmic condition.* The second series were at first looked upon as simply a manifestation of that movable condition of the nervous system known to characterize the patient.

After a few days, the severity of the spasms increasing and the interval between them diminishing, it was concluded that they were of the same character as the initial convulsions. On July 31st, the water was quite bloody, and, although granular and waxy, casts were exceedingly few—cells similar to the white corpuscle of the blood (the exudation cell of George Johnson; the round, germinal matter cell of Beale), were very abundant, occurring in large numbers, and often in clusters, in every field examined.

By the night of August 2nd, very severe paroxysms occurred every ten minutes, while early the next morning they were as frequent as every five minutes. The intervals between a few spasms was as short as two and one-half minutes.

Dr. Bartlett described the convulsions as follows :

The left hand, the fore-arm being semi-flexed on the arm, was observed to make a gentle motion, as if in the act of beckoning ; the next instant the face was drawn powerfully to the left, the mouth drawn violently open and to the left. This state of tonicity soon gave place to one of clonic spasm, affecting all the voluntary muscles. When the paroxysms were severe, there were three stages, viz. : of tonic contraction, of clonic movement, and of paresis. In this latter stage, toward the last, the muscles of the cheeks, jaws, tongue and larnyx lost their power more or less ; respiration was whistling while tonicity endured, and stertorous in the paralytic stage. At times, during the intervals, swallowing was very difficult. Early in the attack the left arm and legs became useless ; the legs seemed paralyzed, and, at the worst, the arm also ; for the most part the muscles of this arm were in a state of half tonic contraction ; later in the attack, both arms were thus affected. During a convulsion, the limbs on both sides were similarly influenced. The pupils were normal during the paroxysms ; in the intervals they were more or less dilated. Sight was usually impaired. Sometimes, before a convulsion, she would speak of a great flame, and then immediately of a horrid darkness. Even in the tonic state of the most violent convulsions, a superficial respiration could be detected by auscultation ; the heart's action was then noted to be regular, though accelerated and enfeebled. During the slighter convulsions, the left side only was affected. The duration of the paroxysms varied from one to two minutes.

On the 11th of August, the disease had reached its worst. Severe convulsions occurred every ten or fifteen minutes ; pulse small, weak and quick, and 145 ; tongue and mouth had a typhoid look ; dysphagia ; mind very dull. Quinine and brandy were given, and inhalation of chloroform continued for forty minutes, and further convulsions prevented by the use of chloroform and chloral, aided by anti-spasmodics and hypnotics. The convulsions were then followed by violent fits of hysterical delirium. This delirium was plainly uræmic, and indicative of less uræmia than the convulsions. It gave place to the delusions of puerperal mania, with unusual hyperæsthesia of certain parts of the surface of the body, which lasted eight days, gradually leaving her with a mind sane and unusally active.

Dr. Bartlett then discussed the nature of the convulsions, and the condition of the patient, during the attacks and in their intervals, and mentioned the various medicines exhibited.

The treatment was directed to the protection of the encephalon; to the control of the spasmodic action by anæsthesia or relaxation, and to the elimination of the toxic elements through the bowels, skin and kidney. For this purpose the following remedies were employed: venesection; chloroform (eight pounds), ether (one gallon), chloral, morphine, bromide of potassium, Indian hemp; lobelia, gelseminum, veratrum viride; croton oil, elaterium, cream of tartar, aconite, citrate of potash, hot baths; colchicum, acetate of potash, and digitalis. Tentatively, quinine and galvanism were used without effect. The patient was well fed throughout. The anæsthetics were generally used upon threatenings of a convulsion, and as far as practicable during the attack.

The active treatment extended *through a period of sixteen days* from the birth of the child, in which time *the patient had at least one thousand convulsions.*

As curiosities of the case, Dr. Bartlett mentioned two features: one, a spindle-shaped swelling, an apparent enlargement of a section of the sterno-cleido-mastoid muscles, about at the junction of the upper with the lower four-fifths, for a distance of an inch and a half. This swelling seemed to change *pari passu* with the œdema. The other peculiarity had reference to the management of the case. The first step in the convulsive phenomena was the turning of the face to the left. Now, when the tendency to spasm was least, the arrest of this movement, the forcible holding of the head from turning to the left, would stay, abort, the convulsion. In the early stage of convalescence, also, delusions would be certain to follow the turning of the face toward the left. The nurse was therefore charged with the duty of keeping the face in the line of the body.

The present condition of the patient is one of incomplete convalescence. Traces of uræmic poisoning still remain. Œdema and slight fainting and sinking attacks, common in Bright's disease, occasionally occur. The urine is now almost free from albumen, and the exudation cells are not numerous.

Dr. Bartlett referred to the hypodermic use of chloral, and said that it produced sloughing of the cellular tissue and integument, unhealthy ulceration and prolonged neuralgic pain.

Dr. Bartlett then called attention to an inhaler used by him in cases where it was necessary to apply chloroform, without the loss of time incident to the ordinary method. It consists of a cup large enough to cover the mouth and nose. Into the bottom of this a concave sponge is held by a transverse stay of wood. The chloroform is poured on the moistened sponge, and the cup is inverted and placed in a shallow vessel, as a saucer, containing water. This arrangement prevents the evaporation of the chloroform, when the inhaler is not in use, so that hours after the anæsthetic is placed on the sponge it may promptly be applied. Dr. Bartlett recommended this inhaler, also, on the score of economy.

Dr. Hamill inquired what was the immediate effect of the powerful remedies, such as gelseminum and veratrum viride?

Dr. Bartlett—The skin became moist; the intervals between the convulsions were prolonged from ten minutes even to an hour. The veratrum acted the best of the relaxing medicines. It tended, however, when long continued, to accelerate the pulse, and make it feeble.

Dr. Hay asked whether it was possible to determine the effect produced on the convulsions by bleeding? In physiological experiments, if an animal be bled suddenly, it will go into convulsions.

Dr. Bartlett—The series of experiments by Richardson on dogs, in which the emulgent veins of the kidney were tied in order to cause an accumulation of urea in the system, showed that, by bleeding one of the dogs, it lived for some time; the one not bled died speedily. In this case a convulsion occurred one hour after the second bleeding.

Dr. Hamill—In the early years of my practice, it was the rule to bleed from both arms at once, and to give jalap and cream of tartar. Whether it is due to the change in woman's body, or of the treatment, more patients recovered in those early days than at the present time.

Dr. Adolphus was of opinion that the convulsions were hysterical, and that tonics and antispasmodics would have formed an excellent line of treatment.

Dr. Freer inquired as to the pathology of the case.

Dr. Bartlett said that, from the outset, the impression was that the convulsions were due to uræmia. The patient is supposed to have Bright's disease.

Dr. Freer—My experience in treating those cases is adverse to the abstraction of blood. Where pathology shows that there is urea in the blood, what good will bleeding do? The effect of urea in the blood, as shown by pathology and physiology, is not that of increasing the force of the blood. It retards the circulation of the blood in the capillaries. There could not be increase of blood pressure in the brain. It is more than probable that the true pathology of uræmia is that of anæmia.

As Dr. Hay has said, it is well known that bleeding will cause convulsions. From a purely scientific stand-point, there is no real ground for bleeding in eclampsia.

Dr. Bartlett—It is excessive bleeding, "bleeding to death," that induces convulsions. The value of venesection in puerperal convulsions was not based on theory, but, as he believed, upon experience. As, in this case, a spasm occurred within an hour after a second bleeding, it could not be claimed that venesection did more than to protect the brain from the effects of the convulsions. In a review of the treatment adopted, certainly no question was more pertinent than the inquiry, What was the effect of the bleeding? Regarding the character of the convulsions, whatever might be their nature, they would have destroyed the patient from their frequency and severity, without treatment.

Dr. Jackson inquired of Dr. Freer, what his treatment has been in such cases?

Dr. Freer—The principal means has been the steady, continual use of chloroform. The case of Dr. Bartlett is not an ordinary one. It is rather like one of hysteria than uræmia, else, unless the patient was different from ordinary women, how could she stand the effects of a thousand convulsions? Active cathartics would relieve the blood of urea, and not impoverish it. Few persons have any too much blood in their systems; they cannot afford to lose this noblest juice of the body. There are other modes of elimination. Probably the convulsions are due to the obstruction of circulation in the brain, the blood not being in a fit condition.

Drs. Hayes, McKennan, James G. Tucker, and Wilder, also engaged in the discussion, of which the notes have been lost or mislaid.

Dr. A. Reeves Jackson gave, verbally, the details of a recent case of chronic vaginitis in a patient fifty-three years of age, illustrative of the efficiency of two means of treatment—injections of hot water, and the use of the glass dilator. Prior to the menopause, there were leucorrhœa, pain, pruritus, and a very irritating, thick, muco-purulent vaginal discharge. There was no uterine disease. The submucous tissue of the vagina was thickened and felt rough. The patient had been subjected to various treatments without relief. A solution of borax had only temporarily relieved the pruritus. Dr. Jackson, after directing the use of injections of glycerine and flax-seed tea for one week, then ordered injections to be given by means of a Davidson or Watson syringe, of from one to two gallons of hot water, beginning with that of a temperature of 98° F. and gradually warming it up to 108° Fahr., repeated daily for two or three days. During the intervals the glass dilator was used. The redness and roughness and discharge disappeared, and the patient fully recovered health.

This treatment separates the walls of the vagina, and so gives rest to the part. Secondly, it lessens the hyperæmia. Thirdly, it causes diminution of the hyperplasia of the submucous tissues.

Where the vaginitis is due to constitutional causes, chlorosis or endometritis, such causes must first be removed. The absence of pain in this mode of treatment makes it a very satisfactory and desirable one. It is beneficial in cervical endometritis. It blanches the surface, lessens the calibre of the capillaries, and contracts the vaginal canal.

Upon motion, the Society adjourned.

